# In silico search for and analysis of R gene variation in primitive cultivated potato species

**DOI:** 10.18699/vjgb-24-21

**Published:** 2024-04

**Authors:** A.A. Gurina, M.S. Gancheva, N.V. Alpatieva, E.V. Rogozina

**Affiliations:** Federal Research Center the N.I. Vavilov All-Russian Institute of Plant Genetic Resources (VIR), St. Petersburg, Russia; St. Petersburg State University, St. Petersburg, Russia; Federal Research Center the N.I. Vavilov All-Russian Institute of Plant Genetic Resources (VIR), St. Petersburg, Russia; Federal Research Center the N.I. Vavilov All-Russian Institute of Plant Genetic Resources (VIR), St. Petersburg, Russia

**Keywords:** R genes, NBS-LRR, polymorphism, Solanum phureja, S. stenotomum, R-гены, NBS-LRR, полиморфизм, Solanum phureja, S. stenotomum

## Abstract

Pathogen recognition receptors encoded by R genes play a key role in plant protection. Nowadays, R genes are a basis for breeding many crops, including potato. Many potato R genes have been discovered and found suitable for breeding thanks to the studies of a wide variety of wild potato species. The use of primitive cultivated potato species (PCPS) as representatives of the primary gene pool can also be promising in this respect. PCPS are the closest to the early domesticated forms of potato; therefore, their investigation could help understand the evolution of R genes. The present study was aimed at identifying and analyzing R genes in PCPS listed in the open database of NCBI and Solomics DB. In total, the study involved 27 accessions belonging to three species: Solanum phureja Juz. & Bukasov, S. stenotomum Juz. & Bukasov and S. goniocalyx Juz. & Bukasov Materials for the analysis were the sequencing data for the said three species from the PRJNA394943 and PRJCA006011 projects. An in silico search was carried out for sequences homologous to 26 R genes identified in potato species differing in phylogenetic distance from PCPS, namely nightshade (S. americanum), North- (S. bulbocastanum, S. demissum) and South-American (S. venturii, S. berthaultii) wild potato species, as well as the cultivated potato species S. tuberosum and S. andigenum. Homologs of all investigated protein-coding sequences were discovered in PCPS with a relatively high degree of similarity (85–100 %). Homologs of the Rpi-R3b, Rpi-amr3 and Rpi-ber1 genes have been identified in PCPS for the first time. An analysis of polymorphism of nucleotide and amino acid sequences has been carried out for 15 R genes. The differences in frequencies of substitutions in PCPS have been demonstrated by analysis of R genes, the reference sequences of which have been identified in different species. For all the studied NBS-LRR genes, the proportion of substituted amino acids in the LRR domain exceeds this figure for the NBS domain. The potential prospects of using PCPS as sources of resistance to Verticillium wilt have been shown.

## Abstract (ru)

Ключевую роль в защите растений от патогенов играют рецепторы, кодируемые R-генами. Они являются генетической основой для селекции многих сельскохозяйственных культур, в том числе картофеля. Множество генов устойчивости у картофеля стало известно и было вовлечено в селекцию благодаря изучению широкого разнообразия диких сородичей картофеля. Использование примитивных культурных видов (ПКВ), относящихся к первичному генофонду картофеля, также перспективно. Как наиболее близкие к ранним доместицированным формам картофеля, ПКВ представляют особый интерес для исследования эволюции генов устойчивости. Целью настоящего исследования стали поиск и анализ R-генов у ПКВ картофеля, геномы которых с различным качеством сборки представлены в базе данных NCBI. Исследовано 27 образцов, относящихся к трем видам: Solanum phureja Juz. & Bukasov, S. stenotomum Juz. & Bukasov и S. goniocalyx Juz. & Bukasov. Проведен in silico поиск последовательностей, гомологичных 26 R-генам, идентифицированных у различных по филогенетической отдаленности от ПКВ картофеля видов: паслёна (S. americanum Mill.), североамериканских (S. bulbocastanum Dunal., S. demissum Lindl.) и южноамериканских (S. venturii Hawkes &Hjert., S. berthaultii Hawkes) диких видов, а также видов культурного картофеля (S. tuberosum L., S. andigenum Juz. & Bukasov). Гомологи кодирующих последовательностей всех исследованных генов обнаружены у ПКВ картофеля с относительно высокой степенью сходства (85–100 %). Впервые у примитивных культурных видов картофеля найдены гомологи генов R3b, Rpi-amr3 и Rpi- ber1. Для 15 R-генов проведен анализ полиморфизма нуклеотидных и аминокислотных последовательностей. Приведены отличия в частоте замен у ПКВ картофеля при анализе R-генов, референсные последовательности которых идентифицированы у разных видов. Для всех изученных NBS-LRR генов доля замещенных аминокислот в LRR-домене превосходит этот показатель для NBS-домена. Показана потенциальная перспективность использования ПКВ картофеля в качестве источников устойчивости к вертициллёзному увяданию.

## Introduction

The main factor in plant evolution is the adaptation to unfavorable external conditions, including microorganisms and pests (Fang et al., 2022). Recently, significant progress has been made in understanding the molecular mechanisms of plantpathogen interaction. It has been established that plants have a multi-level system of protection against pests, including the stages of recognition, signaling and initiation of a protective response (Zhang et al., 2019). Receptors localized on the surface or inside the cell have been shown to play a leading role in the activation of immunity, and R genes (resistance genes) encoding receptors are the genetic basis for breeding many crops for disease resistance (Deng et al., 2020).

In general, plants have two immune systems: PAMP, the pathogen-associated molecular patterns immunity, which includes the recognition of elicitors (conservative non-racespecific signals, such as polysaccharides, chitin, etc.), and ETI, the effector-triggered immunity, which includes the recognition of race-specific effectors. It is with the recognition of effectors that the action of R genes is associated: they directly or indirectly (mediated by other proteins) interact with effectors and trigger the plant immune response, which often includes the programmed cell death that blocks the further spread of the pathogen (Kourelis et al., 2018).

Potato is the most important non-grain crop. In terms of production volume, it ranks fourth among all agricultural crops; according to the FAO data, global potato production in 2020 was over 350 million tons (FAO, 2020). Despite its high adaptive potential, potato is affected by a variety of diseases and pests, 27 of which cause economically significant damage worldwide (Bradshaw, 2021). According to the FAO, potato diseases cause annual losses of about 11.6 % of the gross yield (FAO, 2010). Cultivation of resistant potato cultivars is necessary for stable agricultural production, optimized use of means of chemical control, and for obtaining high-quality products. Introgression of resistance genes from wild and cultivated potato relatives (species of the section Petota Dumort. of the genus Solanum L.) allows the creation of resistant varieties and breeding lines. The search for genes for resistance to pathogens and pests in representatives of different groups of the potato gene pool is a topical trend in research conducted by research centers in Europe, the USA, India and China (Bradshaw, 2021).

The breeding value of tuber-bearing Solanum species depends on their compatibility with cultivated potatoes and the nature of inheritance of the target trait (Rogozina, Khavkin, 2017). Primitive cultivated species, including S. phureja Juz. & Bukasov, S. stenotomum Juz. & Bukasov, S. goniocalyx Juz. &Bukasov (according to J. Hawkes (1990), S. stenotomum subsp. goniocalyx (Juz. & Bukasov) Hawkes), belong to the primary gene pool, representatives of which easily cross with potato cultivars (Bradeen, Kole, 2011). In this regard, the use of primitive cultivated species as source material for breeding is of particular interest.

In 2011, the first article was published describing the genome sequence of the artificially created DM 1-3 516 R44 (DM1-3) doubled monoploid from the group Phureja (Potato Genome Sequencing Consortium (PGSC), 2011). The published sequence represented 86 % of the genome of an artificially created homozygous clone, and was obtained by integrating two assemblies of genomic sequences, i. e. the diploid heterozygous clone RH89-039-16 and the clone DM 1-3 516 R44 (CIP 801092) (The Potato Genome Sequencing Initiative, https://www.hutton.ac.uk/sites/default/ files/documents/posters/sharma/Sharma_Potato_Sequencing_ Initiative.pdf, accessed May 2, 2023).

By 2022, improvements in sequencing technologies made it possible to create assemblies of genomic sequences of representatives of all groups of the potato gene pool, i. e. wild and cultivated species, and tetraploid potato cultivars (Usadel, 2022). Genetic information on potato and related Solanum spp. presented in the database of NCBI, Spud and Solomics DBs contributes to a better understanding of their genetic differences, the process of species evolution, and helps in the search for genes that determine the traits of importance for breeding.

The objective of the present work was the search for and structural analysis of R genes using whole-genome sequencing data, including short-read data (sequence raw archive (SRA)), and genomes partially assembled (to the contig level) for PCPS.

## Materials and methods

Material. The search for resistance genes used the potato reference genome sequence DM1-3 v4.3 (PGSC, 2011), shortread data resulting from the whole-genome sequencing in the PRJNA394943 Project (Li et al., 2018), as well as genome assemblies from the PRJCA006011 Project (Tang et al., 2022) for such PCPS as Solanum phureja, S. stenotomum, and S. goniocalyx (Table 1).

**Table 1.**
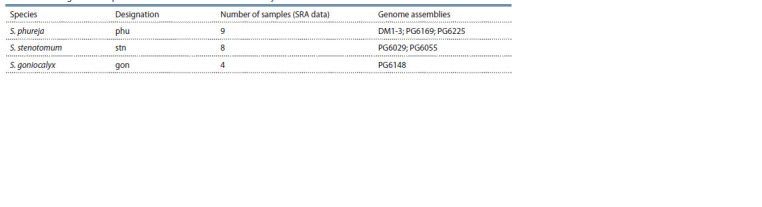
Whole-genome sequences and SRA data included in the analysis Note. Genome assembly identification numbers are given according to the source (Tang et al., 2022). A full description of the material is presented in Supplementary Material 1.

Twenty-six R genes were taken as objects for analysis (the complete list is presented in Supplementary Material 2)1, the reference sequences of which are contained in the database of NCBI. These sequences were isolated from the genetic material of wild and cultivated potato species, as well as American black nightshade S. americanum


Supplementary Materials are available in the online version of the paper: https://vavilov.elpub.ru/jour/manager/files/Suppl_Gurina_Engl_28_2.pdf


In silico search for and analysis of R genes in genomes of PCPS. At the first stage of in silico analysis, a search for R gene homologs was carried out in the whole-genome sequencing data and assemblies of genomes of PCPS.

Search for and analysis of R gene homologs in SRA data. After assessing the quality of sequencing (FASTQC v0.11.9) (Wingett, Andrews, 2018), low-quality reads were filtered (Trimmomatic 0.39). The process included the removal of PCR duplicates, as well as reads containing more than 20 % of nucleotides with Phred quality < 5, or more than 10 % of unidentified nucleotides (Bolger et al., 2014). Further on, the reads of each sample were aligned to the reference R genes using the local multiple alignment algorithm of the bowtie2 v.2.3.5.1 program (Langmead, Salzberg, 2012). The results of alignment were processed using the samtools 1.10 and bedtools 2.30.0 programs; the same programs were used to assess the sufficiency and homogeneity of gene coverage (Quinlan, Hall, 2010). The search for variants was carried out using VarScan 2.4.4 with a minimum coverage of 5 (Koboldt et al., 2009). The results were processed using R 4.2.2 and Python 3.8.2.

Search for and analysis of R genes in genome assemblies for PCPS. The presence of gene sequences in several genome assemblies for PCPS from the PRJCA006011 Project (Tang et al., 2022) was checked using the local blast 2.5.0+ algorithm (Ladunga, 2017). Selection of sequences was based on their similarity to the reference ones (at least 80 %) and at least 80 % coverage of the latter. These sequences could represent both the complete sequences of R genes and their individual parts, i. e. UTRs, exons, and introns. Using ClustalW local alignment, sequences with a fully represented coding region of the gene were selected

At the second stage, resistance genes identified in genomes of PCPS were analyzed for the presence of polymorphism in nucleotide and amino acid sequences. ClustalW local alignment in Mega X software was used to evaluate changes in amino acid sequences (Kumar et al., 2018). InterPro (Paysan- Lafosse et al., 2023) was used to calculate domain organization and key positions in the amino acid sequences of R genes.

## Results

Homologs of all 26 original reference R genes were identified and their significant similarity (over 80 %) in the coding regions was revealed in the whole-genome sequencing data and assemblies of genomes of S. phureja, S. stenotomum, and S. goniocalyx. The non-coding sequences (UTRs and/or introns) in most putative homologs are very different from those in the reference genes, and the degree of their similarity, as a rule, does not exceed 60 %.

The quality and uniformity of SRA data coverage for each of the 26 R genes was assessed, and a search for complete coding sequences in genome assemblies for PCPS was performed. Further analysis excluded genes, the exact and complete protein-coding sequences of which were unknown, as well as genes, the initial search for which demonstrated discrepancies between the data obtained from the study of genome assemblies and SRA data (for instance, the Rpi-mch1 gene had extremely low coverage when SRA data were analyzed, although it was present in the assemblies). Taking into account the published data on the high degree of similarity of genes in clusters on chromosomes IV, VIII and IX, one reference gene was selected from each cluster for analyzing a group of homologs: Rpi-R2-like for the Rpi-R2, Rpi-R2-like, Rpi-abpt, and Rpi-blb3 group of homologs; Rpi-sto1 for the Rpi-sto1, Rpi-blb1, and Rpi-bt1 group; and Rpi-vnt1.3 among different allelic variants of the Rpi-vnt1 gene.

Based on the results of the first stage of analysis, 15 R genes were selected. A number of these genes provide potato resistance to late blight; these are Rpi-R1 (Ballvora et al., 2002), Rpi-R2-like (Lokossou et al., 2009), Rpi-R3a (Huang S. et al., 2005), Rpi-R3b (Li G. et al., 2011), Rpi-sto1 (Vleeshouwers et al., 2008), Rpi-blb2 (van der Vossen et al., 2005), Rpi-vnt1.3 (Foster et al., 2009), Rpi-ber1 (Monino-Lopez et al., 2021), Rpi-R8 (Vossen et al., 2016), and Rpi-amr3 in nightshade (Witek et al., 2021). Also, there was the Rx gene of resistance to potato virus X (Bendahmane et al., 1999), Gpa2 – to pathotype Pa2 of the pale cyst nematode (van der Vossen et al., 2000), Ve1, Ve2 – to verticillium wilt (Song et al., 2017), and Tm2-ToMV – to tomato and tobacco mosaic viruses (Wu X. et al., unpublished). All of these genes are representatives of two families: the Ve1 and Ve2 genes belong to the RLP/ RKL (receptor-like proteins/receptor-like kinases) family, the remaining 13 genes are from the CC-NBS-LRR (Coiled-Coil Nucleotide Binding Site Leucine Rich Repeats) family.

Genome assemblies for PCPS were found to contain different numbers of copies of the analyzed R genes (Table 2). The Rpi-R3a gene is represented by a single sequence in most genomes. The Rpi-amr3, Rpi-R1, Rpi-R3b, Rpi-blb2, Rpi-ber1, Ve1, Ve2, Rx, Gpa2, and Tm2-ToMV genes have from one to 10 copies. More than a dozen copies were found for the Rpi-sto1, Rpi-vnt1.3, and Rpi-R8 genes. The number of copies of the Rpi-R2-like gene in different assemblies can reach 45. No noticeable differences in the number of copies of each gene were observed between genome assemblies; the pairwise correlation coefficient between the number of copies in different assemblies exceeds 80 % (see Table 2).

**Table 2.**
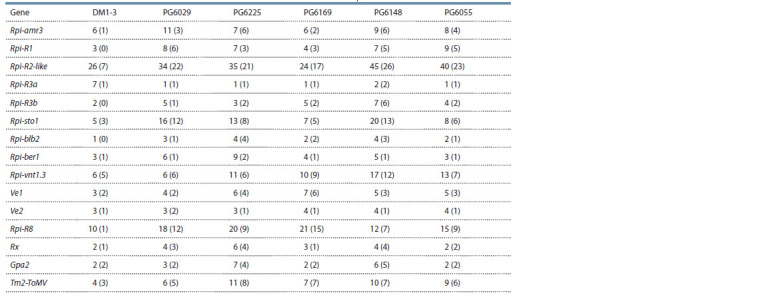
Number of gene copies in assemblies for PCPS, indicating the number of copies that do not contain premature stop codons (in parentheses)

Unlike other assemblies in the reference genome DM1-3, several genes (Rpi-R1, Rpi-R3b, and Rpi-blb2) do not have homologs capable of producing similar proteins (all homologous sequences contain premature stop codons) (see Table 2). We attribute this to the synthetic origin of the doubled monoploid S. phureja.

For the 15 selected reference R genes, nucleotide and amino acid sequences were subsequently analyzed for the presence of polymorphism in PCPS; they were found to contain more than two thousand polymorphism sites in the coding sequences of resistance genes, most of which are single nucleotide polymorphisms (SNPs). Deletions or insertions that affect the predicted amino acid sequence were found in at least one copy of all genes. Nevertheless, variants capable of producing a protein similar to the reference one were found for all genes in the genomes of individual species

**Fig. 1.**
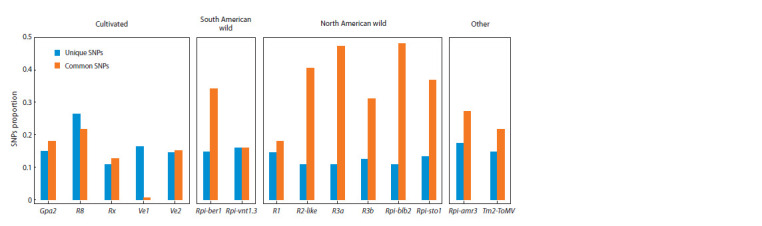
Occurrence of SNPs in R gene homologs among samples of PCPS (according to SRA data).

The distribution of SNP occurrence corresponds to the concept of PCPS as a group of closely related species. Most commonly distributed are the SNPs common for all samples, compared to the unique SNPs found in only one sample. Many common SNPs (572, which is more than 25 % of all detected) distinguished the studied samples from the reference R gene sequences (Fig. 1). Almost 15 % of the detected SNPs were unique sites.

The distribution of SNPs occurrence in samples of PCPS depends on the origin of the source species of the reference R gene. We found significant differences (Kruskal–Wallis test value of 7.4044, p-value = 0.02467) between SNPs common for all studied samples of PCPS in genes from North American wild species and from cultivated potato species (see Fig. 1). We noted similar indicators in the analyzed genome assemblies. The number of variable sites common for PCPS was minimal for the reference genes Rpi-R8, Rx, Ve1, and Ve2, the sources of which are samples of cultivated potato. Meanwhile, more than 40 % of the SNPs detected in the genes Rpi-R3a, Rpi-blb2, and Rpi-R2-like, were the same for all samples of PCPS, the sources of which are samples of North American wild potato species phylogenetically distant from PCPS (see Fig. 1). The differences in the frequencies of unique SNPs are not so pronounced; the largest proportion is characteristic of the Rpi-R8, Ve1, and Gpa2 genes, the sources of which are cultivated potato species

Of the genes originating from North American wild potato species, only the Rpi-R1 gene breaks the trend of a high proportion of common SNPs characteristic of other genes (in this gene, the proportion of SNPs common for PCPS is below 20 %) (see Fig. 1). This gene was introgressed into cultivated potato from a distant wild species S. demissum. However, when examining different copies of this gene present in genomes of PCPS, we found several indels in each of these copies, including deletions up to 15 nucleotides long. We observed a similar decrease in the proportion of common SNPs and the presence of indels in each copy when studying Rpi-amr3, another late blight resistance gene found in S. americanum, one of the nightshade species, also very distant from cultivated potato.

**Fig. 2.**
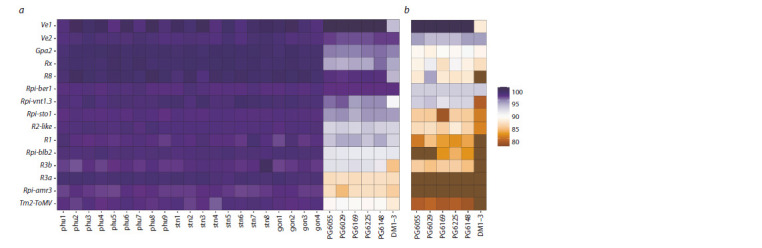
Heat map of similarity of cds nucleotide sequences (a) and amino acid sequences (b) of R gene homologs in PCPS with the reference sequences of R genes. The color scale represents the level of similarity with the reference gene.

An analysis of genome assemblies and SRA data revealed differences in the degree of similarity between sequences found in PCPS and reference sequences of R genes (Fig. 2, a). The differences are most pronounced in multicopy genes. The reason is that the analysis of SRA data actually takes into account the consensus sequence for all copies, while an increase in the number of copies reduces the likelihood of taking into account the SNPs, since the variant that occurs in the majority of copies is taken into account in the consensus sequence. The strongest differences between the estimates of the assembled genomes and raw reads are observed when analyzing the Rpi-R3a and Rpi-amr3 genes (see Fig. 2, a). Most likely, they result from the chimeric alignment of reads from other genes to the reference ones. Further analysis of these sequences did not take into account the results of SRA data processing.

Samples of PCPS showed differences in terms of the degree of similarity of the found homologs (without taking into account the DM1-3 assembly) with the reference sequences of R genes. The median level of differences is 0.5 %. The copies of the Rpi-ber1 and Ve1 genes that are most similar to the reference gene differ in single SNPs (0.01 %) between different genomes of PCPS. The maximum level of differences between the nucleotide sequences of R gene homologs in PCPS was observed for the Rpi-amr3 gene and amounted to 2.3 %. A level of polymorphism close to this value was noted when analyzing Rx, R1, Rpi-vnt1.3, and Rpi-sto1 homologs. It was noted for the Rpi-R8, Rpi-blb2, and Ve2 genes that one or several copies are quite stable and have a low level of polymorphism, while other copies differ significantly from each other. This corresponds to the notions about the evolutionary process of R genes, for which the copy number can reduce the influence of selection

In the assemblies of PCPS (excluding the DM1-3 assembly), homologs of all R genes were found to contain copies potentially capable of producing amino acid sequences (no reading frame shifts or premature stop codons were found in them). The degree of similarity to the reference amino acid sequence varies between 72–100 % (see Fig. 2, b).

The groups of genes, the origin of which is directly related to cultivated potatoes, are clearly divided into two groups based on the similarity of amino acid sequences to the reference ones: the genes of the RLP-RLK family, Ve1 and Ve2, have the highest similarity to the reference gene: 100 and 92–94 %, respectively; the similarity of genes of the CCNBS- LRR family (Gpa2, Rx, Rpi-R8) is significantly lower 85–90 % (see Fig. 2, b). The genes from the CC-NBS-LRR family, the origin of which is associated with South American wild species, have a higher amino acid sequence similarity to the reference ones than the Rpi-R2-like, Rpi-sto1, Rpi-R1 and Rpi- R3b genes from North American wild species: about 95 and 80–85 %, respectively. Changes in the nucleotide sequence in the Rpi-R3a, Rpi-amr3 and Tm2-ToMV genes lead to low similarity (75–80 %) with the reference amino acid sequence (see Fig. 2, b).

The functional analysis of amino acid sequences performed using InterPro showed that the homologs of the NBS-LRR genes in PCPS have no changes in domain organization, even in the sequences, the similarity of which to the reference sequence is below 80 %.

**Fig. 3.**
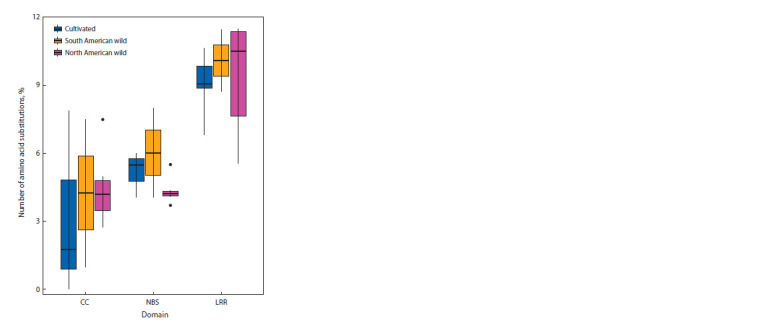
Comparison of the proportion of amino acid substitutions in different domains of R genes. Significant differences between LRR and other domains were assessed according to the Kruskal–Wallis test value of 17.343, p-value = 0.0001714

The distribution of amino acid substitutions across individual domains in genes of the CC-NBS-LRR family is shown in Fig. 3. Only sequences forming complete proteins (free from premature stop codons) were taken into account. The Ve1 and Ve2 genes, which, according to InterPro and NCBI data, have a different domain organization compared to CC-NBS-LRR, are not included.

The percentage of substituted amino acids varies significantly depending on the domain (see Fig. 3). The LRR domain in all found R gene homologs demonstrated the largest number (on average more than 9 %) of amino acid substitutions compared to the reference. In the Rpi-vnt1.3, Rpi-sto1, Rpi-R1, and Rpi-R3b genes, the proportion of amino acids substituted in the LRR domain exceeds 10 %. The proportion of substitutions in the CC domain varies significantly between genes. This domain was found to contain no amino acid substitutions in homologs of the R8, Rpi-vnt1.3, and Rx genes, while there were 7 % amino acid substitutions in Rpi-blb2 and Gpa2, and 11 % in the Tm2-ToMV gene. The NBS domain contains about 5 % substituted amino acids on the average.

The distribution of substituted amino acids in different domains among PCPS is also uneven. In the CC domain of almost all genes, most substitutions occur only in individual samples or small groups. Substituted amino acids common for all samples of PCPS are represented by 0–3 sites. An exception is the Gpa2 gene, in which five substitutions in the CC domain are common, and several of them are located either directly in the RanGAP2 interaction site (according to NCBI data), or in close proximity to it (neighboring amino acids). In the NBS domain, substitutions common for PCPS are prevalent, and Rx was the only gene where they are absent. In the LRR domain, amino acid substitutes that are common or characteristic only of some samples are distributed relatively evenly.

## Discussion

Potato is a heterozygous tetraploid, the genetic analysis of which is a challenge. Genomic studies of potatoes and related tuber-forming species, the search for and annotation of genes that determine agronomically important characteristics make a significant contribution to genetics proper of the crop and the improvement of breeding technologies (Bradshaw, 2021). PCPS belong to a systematic group of interest from the evolutionary point of view and as a source of important potato traits of value for breeding, such as disease resistance.

We conducted a bioinformatics analysis of whole-genome sequencing data from 27 samples of S. phureja, S. stenotomum and S. goniocalyx (21 from SRA data and 6 genome assemblies) for 26 genes of resistance to pathogens and pests. All samples contained sequences homologous to the coding regions of all analyzed R genes, and their significant similarity (> 85 %) with the corresponding regions of reference genes was revealed. The noncoding sequences (UTRs and/or introns) in most putative homologs differed greatly from those in the reference genes, and the degree of their similarity, as a rule, did not exceed 60 %.

Many of the reference sequences were obtained from species distant from PCPS. For instance, the Rpi-amr3 gene sequence was isolated from the nightshade S. americanum, and Rpi-blb2, from S. bulbocastanum, a representative of the North American wild potato species. The latter does not directly cross with cultivated species, and the discovery of R gene homologs with a fairly high level of similarity in PCPS is of interest from the point of view of peculiarities of these genes’ evolution. For the first time, homologs of the Rpi-R3b, Rpi-amr3, and Rpi-ber1 genes were discovered in S. phureja and S. stenotomum

Cultivated potatoes grown in South America were found to contain homologs of genes providing reliable protection against late blight, which had been found in the South American wild species S. venturii and the Mexican species S. bulbocastanum and S. stoloniferum, as well as homologs of race-specific late blight resistance genes from the North American species S. demissum. Previously, molecular genetic screening of accessions from the VIR potato collection revealed the presence of SCAR markers of the Rpi genes from wild potato species of the North and South American series, and in samples of PCPS (Muratova et al., 2020; Gurina et al., 2022; Rogozina et al., 2023). The similarity of wild and cultivated potatoes in genes of resistance to the highly specialized pathogen Phytophthora infestans (Mont.) de Bary confirms the conclusion about the inheritance of part of the genetic material from wild species by diploid cultivated potatoes (Hardigan et al., 2017).

The discovery of not only genes protecting potatoes from late blight, viruses, and pale nematodes, but also homologs of R genes from tomato and nightshade in representatives of cultivated potatoes, is in good agreement with the data on a significantly larger repertoire of disease resistance genes in potatoes compared to the closely related Solanaceous crops (Tang et al., 2022).

The previously conducted bioinformatics analysis of the potato reference genome DM1-3 (Jupe et al., 2012; Lozano et al., 2012) was performed without taking into account the R3b, Rpi-amr3, Ve1, Ve2, and Rpi-ber1 genes, since their sequences were not yet known. In a later work devoted to the search for NBS-LRR genes in the cultivated potato S. stenotomum subsp. goniocalyx, the R8, Ve1, Ve2, Rpi-ber1, and Tm2-ToMV gene sequences were not used (Liu, 2020). For the majority of the R gene homologs that we identified, the degree of their similarity to the reference sequences corresponds to the published data (Lozano, 2012; Liu, 2020). However, in the studied samples of S. goniocalyx, sufficiently reliable homologous sequences for the Rx, R3b, and Gpa2 genes were not found (Liu, 2020). Most likely, this is due to the extremely high degree of polymorphism in the non-coding parts of these genes, which, moreover, occupy rather extensive areas, which prevents their full-length analysis

The diploid species S. phureja and S. stenotomum are the closest to the early domesticated forms of tuber-forming species of the genus Solanum L. Representatives of these species were found to contain homologs of R genes with different evolutionary histories. The ancient origin of the extracellular receptor encoded by the Ve1 gene is indicated by the wide distribution of functional and nonfunctional Ve1 homologs in plants of the Solanacea family and other phylogenetically distant species (Song et al., 2017). In PCPS, copies of the Ve1 gene were found in all assemblies, the amino acid sequences of which do not differ from the reference, thus indicating prospects of this group as a potential donor of resistance to Verticillium wilt.

The evolution of Rpi-blb1 and Rpi-blb3, the genes of the Mexican species S. bulbocastanum, proceeded at different rates and in different ways, and only sequences identical to the reference gene were found in S. bulbocastanum samples for the Rpi-blb2 gene (Lokossou et al., 2010). Therefore, the discovery of Rpi-blb2 and Rpi-sto1 homologs (Rpi-blb1 ortholog) in cultivated potatoes growing on another continent and non-crossable with S. bulbocastanum is of particular interest.

As a rule, R genes, especially representatives of the NBSLRR family, are presented in the genome not singly, but in clusters, which are numerous copies of homologous genes (Prakash, 2020). In genomes of PCPS, most of the studied R genes (except for R3a, Rpi-ber1, and Ve2) are also represented by two or more copies. The R2-like gene, which belongs to a large cluster of R genes located on the fourth chromosome, especially stands out. Its copy number among PCPS was the highest, and the number of identified copies varied from 24 to 40. According to the literature, the presence/ absence of a gene and its copy number is a common type of polymorphism among resistance genes, since their organization contributes to unequal recombination, which results in differences in the number of copies of one gene. For some genes, we did observe differences in copy number, but since the assemblies are incomplete, and the method only showed homologs with similarities above the threshold, we cannot say for sure that the revealed differences really occur instead of being a consequence of methodological aspects of the work.

Many authors associate the process of domestication with the loss of diversity in many genes, primarily the economically valuable ones, which include R genes (Li Y. et al., 2018; Tang et al., 2022). Y. Li et al. (2018) noted lower variability in the genetic material of cultivated species compared to wild ones. This agrees well with the high proportion of common SNPs in PCPS, as well as with the potato taxonomy developed by D. Spooner, who considers the species of this plant as a group within one species S. tuberosum (Spooner et al., 2014).

The R genes under study have a complex structure, and the unequal rate of amino acid substitutions in different domains has been previously shown (Prakash et al., 2020). For instance, the NBS domain is more conservative, since it is associated with the activation of the plant protection mechanism inside the cell, while the LRR domain responsible for pathogen recognition is more variable, and the rate of substitutions in it is higher. The uneven distribution of amino acid substitutions across the domains of NBS-LRR proteins discovered in our research corresponds to literature data on the rate of these proteins evolution.

In terms of the prevalence of substituted amino acids among PCPS samples, the R gene domains are also not uniform. The most widespread are changes in the NBS domain, which are common for this group, and are likely associated with changes in proteins subsequently activated by R genes. On the contrary, the CC domain has the smallest number of amino acid substitutions common for PCPS. Little is understood about the function of this domain; in some proteins, it is known to trigger the mechanism of cell death (Huang J. et al., 2021), but for other genes, its interaction with the pathogen recognition system has been shown (Rairdan et al., 2008). It is quite difficult to guess what causes such differences in PCPS and what they can lead to, but when studying different copies, we found that indels were located exactly in the CC domain in the majority of clearly non-functional sequences (containing a premature stop codon). This may reflect the evolutionary nature of diversification of paralogs in the genome while a relatively stable variant is maintained

The LRR domain was found to contain substituted amino acids, both common ones and those characteristic of individual samples or large groups; most likely, these substitutions are part of the mechanism of PCPS ancestral forms adaptation to the races of pathogens common in those times. The mechanism of the LRR domain operation is poorly understood. It has been shown that even a small number of changes in specific amino acids can result in a change in pathogen recognition from virus to nematode (Slootweg et al., 2017). At the same time, large groups of orthologs that recognize the same pathogen are known to be only 85–90 % similar to each other (Park et al., 2005). Apparently, only changes in specific patterns in the sequence lead to changes in the effector, which is recognized by the LRR domain. A detailed study of this phenomenon, however, requires a significantly larger number of well-phenotyped samples, as well as the use of methods that allow the assessment of the interaction of products of different genes.

## Conclusion

Primitive cultivated potato species were found to contain sequences homologous to R genes that provide protection of potatoes from late blight, viruses, potato cyst nematode, of potatoes and tomatoes from Verticillium wilt, and of tomatoes from mosaic viruses. For the first time, a search was carried out and showed the presence of homologs of the R3b, Rpi-amr3, Ve1, Ve2, and Rpi-ber1 genes in PCPS. The similarity of the coding sequences found in PCPS to the reference R genes is over 85 % for all genes. It was shown for the first time that homologs of all R genes in all the studied genome assemblies have copies, among which all genomes except DM1-3 have at least one sequence that does not contain stop codons or frameshifts.

The studied group of samples showed differences in the nature of R gene polymorphism, depending on the source of the reference gene. The changes characterizing the PCPS group as a whole are significantly more represented for homologs of genes from North American wild species, compared to genes from cultivated potato species. It was shown for the first time that one of the copies of the Ve1 gene in PCPS does not contain amino acid substitutions relative to the reference gene, which indicates the potential resistance of PCPS to Verticillium dahliae Kleb.

## Conflict of interest

The authors declare no conflict of interest.
